# Supplementary figures and images for: Protocol development for discovery of angiogenesis inhibitors via automated methods using zebrafish
Source: PLoS One. 2019 Nov 15;14(11):e0221796. doi: 10.1371/journal.pone.0221796 (PMC6857904; doi:10.1371/journal.pone.0221796)

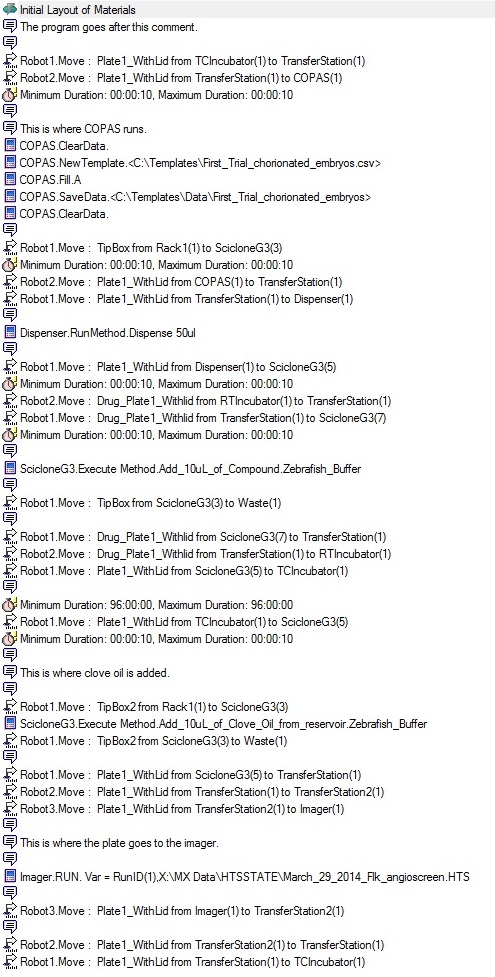

Supplement: S1 Fig — The method begins by initializing all the required equipment and confirming that all plates and consumable are programed in a logical location. As the robots place the 96-well sample plates on the device corresponding to a certain protocol step a separate routine, program, protocol, or simple instruction is triggered by iLink and run by the device. When the device is done working through the set of commands, it triggers iLink to move onto the next step. (JPG) [file pone.0221796.s001.jpg]

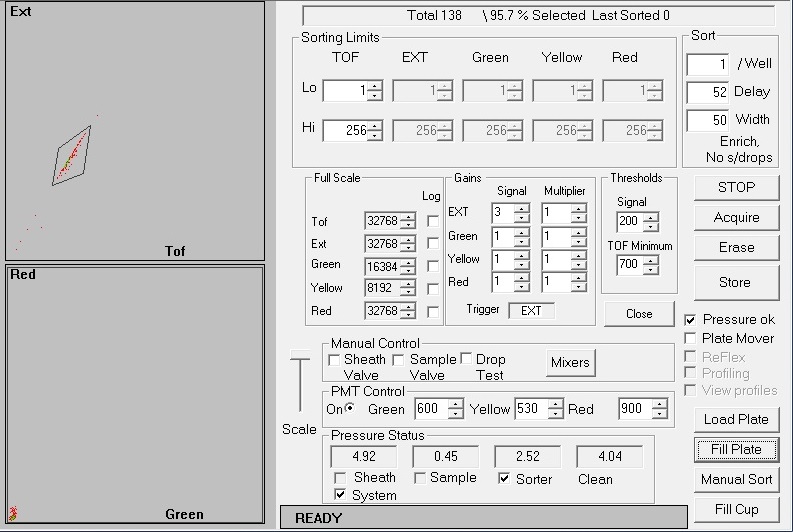

Supplement: S2 Fig — (JPG) [file pone.0221796.s002.jpg]

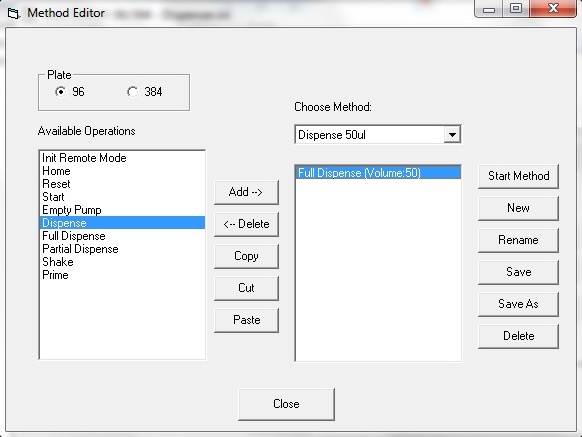

Supplement: S3 Fig — (JPG) [file pone.0221796.s003.jpg]

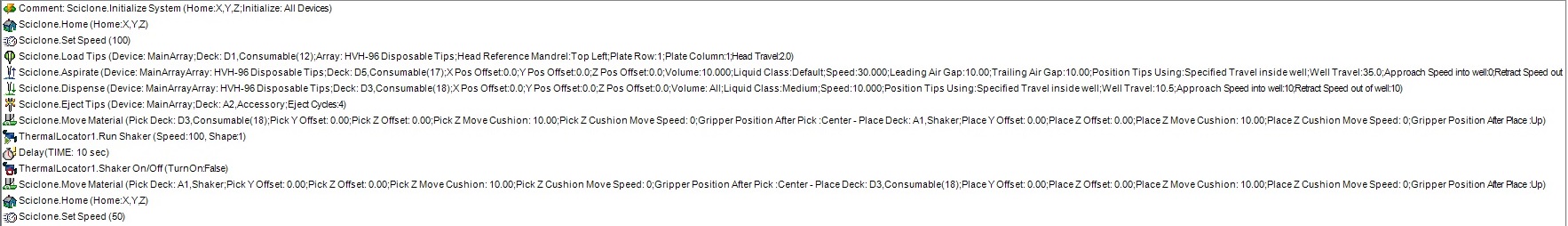

Supplement: S4 Fig — (JPG) [file pone.0221796.s004.jpg]
