# Supplementary material for: Protocol development for discovery of angiogenesis inhibitors via automated methods using zebrafish
Source: PLoS One. 2019 Nov 15;14(11):e0221796. doi: 10.1371/journal.pone.0221796 (PMC6857904; doi:10.1371/journal.pone.0221796)
Supplement: S1 File — (PDF) [file pone.0221796.s007.pdf]

Average Pixel Counts

	Control (0.05% DMSO)	1 µM	2 µM	4 µM	8 µM	16 µM
Plate1	28018.25	27097.5	26518.31	25765.54	18656.64	16119
Plate2	24662.86667	24120.92	23848.07	25678.13	21545.33	18154
Plate3	25203.30769	25480.15	26555.46	23109.36	19561.5	20135.89

	Control (0.05% DMSO)	1 µM	2 µM	4 µM	8 µM	16 µM
mean of averages	25961.47479	25566.19	25640.61	24851.01	19921.16	18136.3
sd	1801.599938	1490.153	1552.501	1508.946	1477.551	2008.503

Dose Response

µM	0	1	2	4	8	16
Plate1 %	25	0	18.75	18.75	26.67	35.71
Plate2 %	0	18.75	6.25	0	20	43.75
Plate3 %	18.75	18.75	13.33	12.5	20	43.75

µM	0	1	2	4	8	16
mean of % dead	14.58333333	12.5	12.77667	10.41667	22.22333	41.07
sd	13.0104125	10.82532	6.268344	9.547033	3.850926	4.641896

µM	0	1	2	4	8	16
Plate1 %	75	100	81.25	81.25	73.33	64.29
Plate2 %	100	81.25	93.75	100	80	56.25
Plate3 %	81.25	81.25	86.67	87.5	80	56.25

µM	0	1	2	4	8	16
mean of % alive	85.41666667	87.5	87.22333	89.58333	77.77667	58.93
sd	13.0104125	10.82532	6.268344	9.547033	3.850926	4.641896
	Control (0.05% DMSO)	1 µM	2 µM	4 µM	8 µM	16 µM
